# Vortex Polymer Optical Fiber with 64 Stable OAM States

**DOI:** 10.3390/polym12122776

**Published:** 2020-11-24

**Authors:** José A. Borda-Hernández, Claudia M. Serpa-Imbett, Hugo E. Hernandez Figueroa

**Affiliations:** 1 Department of Communications, School of Electrical and Computer Engineering, University of Campinas, Campinas, SP 13083-852, Brazil; jbordah@uni.pe (J.A.B.-H.); hugo@decom.fee.unicamp.br (H.E.H.F.); 2 ITEM Research Group, Department of Electronic Engineering, Universidad Pontificia Bolivariana Sede Monteria, Km 6 Via Cerete, Monteria, Córdoba 230002, Colombia

**Keywords:** vortex fibers, CYTOP, polymer optical fiber, multimode fiber, orbital angular momentum

## Abstract

This research introduces a numerical design of an air-core vortex polymer optical fiber in cyclic transparent optical polymer (CYTOP) that propagates 32 orbital angular momentum (OAM) modes, i.e., it may support up to 64 stable OAM-states considering left- and right-handed circular polarizations. This fiber seeks to be an alternative to increase the capacity of short-range optical communication systems multiplexed by modes, in agreement with the high demand of low-cost, insensitive-to-bending and easy-to-handle fibers similar to others optical fibers fabricated in polymers. This novel fiber possesses unique characteristics: a diameter of 50 µm that would allow a high mechanical compatibility with commercially available polymer optical fibers, a difference of effective index between neighbor OAM modes of around 10^−4^ over a bandwidth from 1 to 1.6 µm, propagation losses of approximately 15 × 10^−3^ dB/m for all OAM modes, and a very low dispersion for OAM higher order modes (±*l* = 16) of up to +2.5 ps/km-nm compared with OAM lower order modes at a telecom wavelength of 1.3 µm, in which the CYTOP exhibits a minimal attenuation. The spectra of mutual coupling coefficients between modes are computed considering small bends of up to 3 cm of radius and slight ellipticity in the ring of up to 5%. Results show lower-charge weights for higher order OAM modes.

## 1. Introduction

Mode division multiplexing (MDM) has been used as an alternative to increase the capacity of long-length fiber-optic communication systems through the transmission of modes as independent channels over silica multimode fibers, such as few-mode fibers (FMF) [1], and vortex fibers, which are few-mode fibers designed to lift the near-degeneracy between orbital angular momentum (OAM) modes, minimizing modal crosstalk between them [2]. Moreover, MDM is also an alternative for the consolidation, in a cost-effective manner, of short-range optical communication systems deployed in data centers, in-home, and in-building, among others, in which fiber-optic cables are becoming dominant due to their higher bandwidth and smaller light weight and size compared to twisted pair copper cables [3]. However, silica fibers for MDM are still an expensive choice; furthermore, they lose power when deployed around small bends, such as in corners and staples of the short-range cabling [4].

Another option is the multimode polymer optical fiber (POF) that, compared to multimode and single-mode silica optical fibers, offers several advantages over short distances less than 1 km, such as lower cost and an easier splicing and connecting as well [4,5,6,7,8]. Multimode POFs are used for data transmission in short-range scenarios as well as low-cost optical sensors. High attenuation and low bandwidth over telecom wavelengths still limit transmission distances and capacity. Currently, single-mode bend-insensitive fibers (BIFs), fabricated in silica, and multimode POFs have been proposed as alternatives to be deployed in staples and corners [9]. However, the low bandwidth of up to 2 Gbps of multimode POFs compared with the 10 Gbps of single-mode fibers limits their use in communication systems to support bandwidth-hungry applications [10,11]. To increase the capacity in POFs, a novel design of a multimode POF is proposed, named, henceforth, vortex-POF, to enable the multiplexing and stable propagation of modes with orbital angular momentum (OAM) as independent channels, increasing its capacity in this way. Vortex-POFs would become another option of high capacity compared with single-mode fibers.

Vortex fibers [12] were conceived to stably propagate OAM modes in long fiber lengths, as well as to be mechanically and modally compatible with standard single-mode fibers (SMFs), even enabling simultaneous operation of systems multiplexed by wavelengths (WDM) and multiplexed by modes (MDM) [2,13,14,15]. However, there have had no reports of OAM transmission focused in short-range scenarios, such as data centers [16], with fiber lengths less than 100 m using fibers with materials other than silica.

OAM is another dimension for modal multiplexing. The orthogonality amongst OAM modes that remains almost unchanged even under great perturbations, such as small bends and twists in fibers [15], and its theoretically infinite states associated with the topological charge ±4, makes this kind of multiplexing an excellent alternative to increase transmission distances or capacity. OAM propagation has been widely studied in free space [17,18,19,20], in silica vortex fibers [2,13,14,15,21,22,23,24,25] and in vortex-POFs [26,27]. However, the new design presented possesses unique characteristics: the preservation of both the core/cladding material cyclic transparent optical polymer (CYTOP) and a diameter of 50 µm that is matched to the specifications of commercially available graded index polymer optical fibers (GI-POFS) [28], i.e., a good mechanical compatibility between a POF butt-coupled with our designed vortex-POF, promoting, in this way, easy splicing and connection and maximizing the power coupling between them. These peculiarities allow this design a better performance compared with the previous one reported in [27], allowing even operation further than visible wavelength. This new design shows the alternative of an air-core surrounding a ring of high refractive index in order to allow high refractive index contrast between the modes’ transmission region into the ring and cladding to ensure the transmitting OAM modes not to couple into *LP (linearly polarized)* modes [15]

This study found out that the vortex-POF can support up to 64 stable OAM states, i.e., 32 OAM modes of orbital charge ±*l* with circular right- or left-hand polarizations. The difference of effective index between neighbor OAM modes is around 10^−4^ over a supra-high bandwidth from 1 to 1.6 µm, enough for the stable propagation of OAM modes at distances of up to 1 km. This proposed vortex-POF possesses a propagation loss of approximately 15 × 10^−3^ dB/m for all OAMs, almost the same of bulk material losses, so the propagation loss of each mode can be considered negligible, and a very low dispersion of up to +2.5 ps/km-nm for the highest order OAM modes ±*l* = +16, at a telecom wavelength of 1.3 µm, in which the CYTOP has a minimal attenuation [5]. In addition, the OAM modes of this vortex-POF are very insensitive to coupling by perturbations, such as small bends and slight ellipticity in the ring. Numerical results show that the charge weights defined as the square of the coupling coefficient in dB units are quite low (<−40 dB) for higher order modes under small bends of up to 3 cm of radius and changes in the circularity of up to 5%.

## 2. Theory

### 2.1. OAM Modes in Optical Fibers

The OAM modes in fiber conform to an orthogonal basis that comes from hybrid modes (*HE_l_*_+1,*m*_/*EH_l_*_−1,*m*_) of the conventional step-index optical fiber [29]:

(1) $$HE_{l+1,m}^{even,odd}=F_{l,m}\left(r\right)\left\{\begin{array}{c} \hat{x}\cos l\phi -\hat{y}\sin l\phi \\ \hat{x}\sin l\phi +\hat{y}\cos l\phi \end{array}\right\}e^{ik_{z,HE}.z}$$

(2) $$EH_{l-1,m}^{even,odd}=F_{l,m}\left(r\right)\left\{\begin{array}{c} \hat{x}\cos l\phi +\hat{y}\sin l\\ \hat{x}\sin l\phi -\hat{y}\cos l \end{array}\right\}e^{ik_{z,EH}.z}$$

where *F_l_*_,*m*_(*r*) is the mode’s electric field envelope, *m* is the radial mode number, *m* − 1 the number of zeros in *F*, and *k_z_* is the longitudinal wave vector of the mode related to the effective index given by *k_z_* = 2π*n_e f f_/*λ.

*HE_l_*_+1,*m*_/*EH_l_*_−1,*m*_ even and odd solutions are degenerate with each other in conventional circular fibers with a step index profile. However, *HE_l_*_+1,*m*_ and *EH_l_*_−1,*m*_ are not degenerate, which means *k_z_*_,*HE*_ Ç *k_z_*_,*EH*_. Hybrid modes are not used as independent channels in a fiber-optic-based communication system because they are easily coupled among them under perturbations; moreover, it is very difficult to excite them due to their polarization distribution is spatially varying [15].

The OAM modes in fiber come from a specific linear combination of degenerate pairs (even and odd solutions) of hybrid modes *HE^even^*^,*odd*^ or *EH^even^*^,*odd*^ with π/2 phase shift among them [21]:

(3) $$V_{l,m}^{\pm }=HE_{l+1,m}^{even}\pm iHE_{l+1,m}^{odd}=\hat{\sigma^{\pm }}F_{l,m}\left(r\right)e^{\pm il\phi }e^{ik_{z,HE}.z}$$

(4) $$W_{l,m}^{\pm }=EH_{l-1,m}^{even}\mp iEH_{l-1,m}^{odd}=\hat{\sigma^{\pm }}F_{l,m}\left(r\right)e^{\mp il\phi }e^{ik_{z,EH}.z}$$

where $\hat{\sigma^{\pm }}=\hat{x}\pm i\hat{y}$ indicates left- and right-handed circular polarizations, *l* = ±4 being the state of topological orbital charge of OAM mode. This linear combination exhibits an azimuthal dependence of the phase *e* ± *i*4φ and uniform polarization σˆ making the modal excitation easier compared to hybrid modes. Equations (3) and (4) show that hybrid modes in fiber are combined to produce OAM modes, with *l* = ±*l* for $V_{l,m}^{\pm }$ and *l* = ∓*l* for $W_{l,m}^{\pm }$ resulting in four states [15].

### 2.2. Modal Coupling in Multimode Optical Fibers

Modes in an ideal optical fiber form an orthogonal basis. An optical fiber has, ideally, a circular cross-section, and no changes are assumed in the fiber cross-section along the longitudinal axis (*z*). However, in a real fiber, the refractive index profile is perturbed by diameter-induced variations and inhomogeneity in the material during the fabrication processes. Similarly, external perturbations, such as fiber bends and twists, may change the field distributions of the modes, leading to a coupling among them, which leads to a loss of orthogonality [29], yielding an unstable propagation of the OAM modes at a specific propagation length.

There are two ways to assess the stability of a propagated OAM mode. One is through effective index separation, which has had wide experimental confirmations, providing a degree of reliability. The other is based on the *OAM charge weight* that depends on the electric field overlap between the modes of interest, the form, the symmetry, and the strength of the perturbation on the fiber [30].

#### 2.2.1. Effective Index Separation

Effective index separation depends on the waveguiding size and the index contrast between the core and cladding [30]. In multimode fibers (MMFs), higher order modes belonging to a “*mode group*” have a very low effective index separation, and an easy coupling caused by the perturbations along the longitudinal axis (*z*) is produced [31]. It has been demonstrated that perturbations along the longitudinal axis (*z*) produce distributed mode coupling that can be modeled through a factor inversely proportional to ∆*n*^−*p*^, with *p* ≥ 4 which allows for achieving a stable propagation for a length scale exceeding 100 m [32]. Likewise, by adopting this criterion, the higher order modes belonging to the same “*mode group*”, considered as “*almost degenerates*”, should be stably propagated in a scale of a similar length, as long as ∆*n_e f f_* ≥ 10^−4^ [13]. Stability means both no coupling and no change in the electric field distribution of each individual mode at a specific propagation length.

#### 2.2.2. OAM Charge Weight

Another way to quantify how a perturbation changes the modal coupling consists of obtaining the Ci coupling coefficients that form a set called OAM charge weight distributions or OAM Spectra [33]:

(5) $$C_{i}=∯E\left(x,y,z\right)\psi_{i}^{*}\left(x,y,z\right)dxdy$$

where *C_i_* arises by computing the overlap integral among the components of the normalized electric field *E* (*x*, *y*, *z*) of a mode in the presence of perturbations, multiplied by the normalized electric field ψ*_i_*(*x*, *y*, *z*) of the *i*-eigenmodes in an ideal unperturbed fiber. *C_i_* quantifies the “*mode purity*”, or the ability of a perturbed mode to remain coupled with itself and uncoupled with other modes, where |*C_i_*|^2^ is the *OAM charge weight* or 10log(|*C_i_*|^2^) in dB units and $\sum^{\;}{\left|C_{1}\right|}^{2}=1$. 

This work numerically characterizes the vortex-POF under perturbations, such as bends and changes in the circularity of fiber rings. Fields resulting after bends are modeled using the approach proposed by [34], known as *transformation optics* (TO), to numerically compute the fields in bent fibers.

### 2.3. Fibers for OAM Propagation

**Figure 1:**
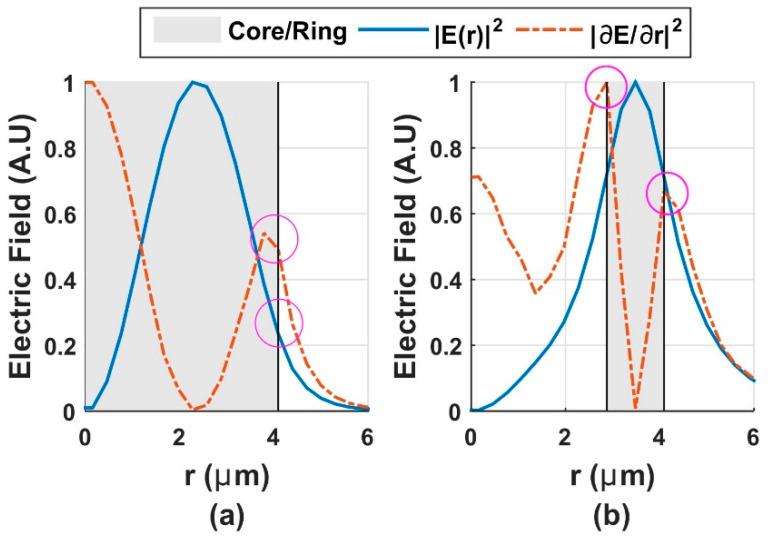
Normalized index profile (gray background) and corresponding mode intensity |*E*(*r*)|2 and its derivate for the scalar *LP*11 mode; (**a**) conventional step-index fiber and (**b**) the ring design.

In a conventional fiber with a step-index profile, a “*group of modes*” is formed by those neighbor modes with a quite near-effective index, such as the *HE_l_*_+1,*m*_ and *EH_l_*_−1,*m*_ modes, and sometimes, *TE*_0*m*_ or *TM*_0*m*_ are included as well. Under weakly guiding approximation that leads a scalar solution of Maxwell equations, vector modes of the same “*group of modes*” become linearly polarized *LP* modes (*LP_lm_* modes) [29], preventing a stable propagation of vector modes in long lengths. However, there is a waveguide developed in [35] and widely adopted to lift the degeneracy among vector modes. This consists of a design whose profile mirrors that mode itself, producing a high mode intensity and its derivate close to the waveguide transition regions (boundaries). These have a ring index profile that yields a large degeneracy between the vector modes of a group. Figure 1 shows an example using the solution of the *LP*_11_ mode of a single-mode fiber with the conventional step-index profile (Figure 1a), and in a vortex fiber with the index profile (ring design) proposed (Figure 1b).

Circles in Figure 1 indicate the field intensity (solid line) and its derivate (dotted line) on the interface, so higher values are for the ring design presented in Figure 1b allowing to lift the degeneracy of the “*group of modes*”. With this proposed index profile of ring design, the index profile of vortex fibers are sculpted, enabling the stable propagation of OAM modes in long lengths [15,22,33,36,37,38,39,40].

The *air-core design* [15] is particularly adopted since it has shown a highly effective index separation among highest order OAM modes in silica fibers and has also shown to be very insensitive to coupling OAM modes under great perturbations, such as small bends.

## 3. Vortex-POF

Our proposed vortex-POF was designed following the criterion depicted in the previous section. We chose a polymer material named CYTOP, used in the fabrication of commercial multimode graded index polymer optical fibers (GI-POFs) that operate at a telecom wavelength near to 1.3 µm.

**Figure 2:**
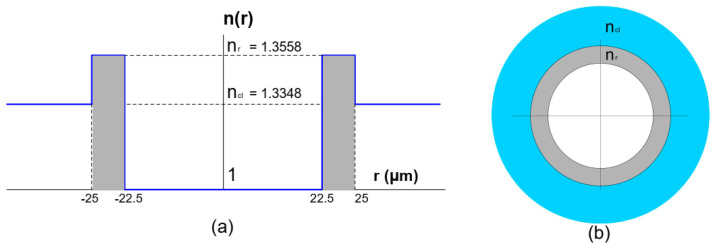
(**a**) Index profile of the proposed vortex polymer optical fiber (POF) by adopting an air-core design. *n_r_* is the refractive index of the ring and *n_cl_* is the cladding refractive index. (**b**) Cross-section of the designed fiber.

With an attenuation of 15 dB/km [5], which is the minimum around this wavelength, we adopted an air-core design as shown in Figure 2.

In order to allow a high mechanical compatibility with GI-POF, this vortex-POF was designed with a core size of *r_core_* = 25 µm [28] and a ring size of ∆*r* = 2.5 µm. First, a modal analysis in COMSOL Multiphysics 5.1 was implemented, which provides a full-vector finite element method to compute the effective index and the electric field distribution of the modes. The refractive index structure was also implemented, shown in Figure 2 surrounded by a perfectly matched layer, which was adapted to the cladding region as an absorbing boundary condition, for leaky waves to be radiated out of the fiber without reflections on the borders of the simulation space. This proposed vortex-POF was simulated considering a telecom wavelength of λ *=* 1.3 µm, which implies a cladding refractive index of *n_cl_* = 1.3348 [41], a contrast between the refractive index in the core and cladding of ∆*n* = 0.021 and an extinction coefficient of *k* = 3.573 × 10^−10^ [41,42]. Additionally, we implemented a sweep of wavelengths from 1 to 1.6 µm to obtain the effective index of OAM modes as a function of light wavelength to verify the modal cut-off of each propagated mode.

## 4. Results

### 4.1. OAM Modes in the Vortex-POF

**Figure 3:**
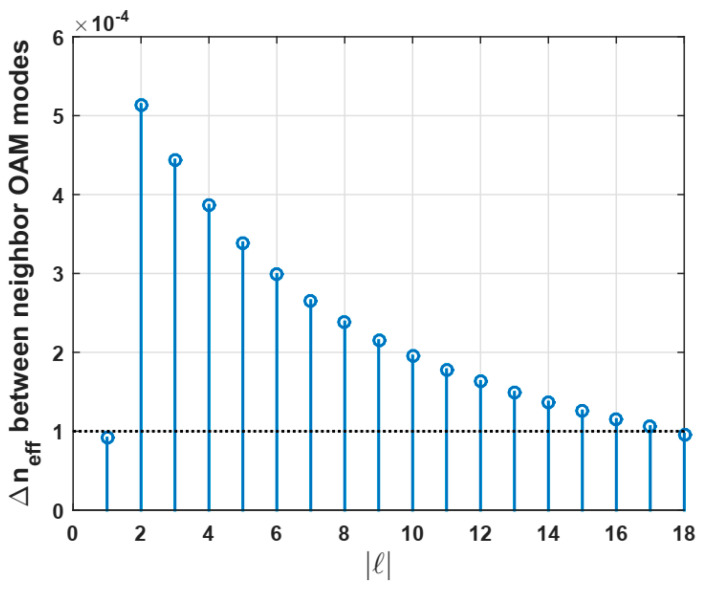
Splitting of *ne f f* between neighbor OAM modes at a telecom wavelength of λ = 1300 nm.

Up to 38 propagated modes were found: the fundamental mode *HE*_11_ with two polarizations, *TE*_01_, *TM*_01,_ and *HE*_21_, the modes with *m* = 1 (with only the first radial order) from *HE*_31_ to *HE*_19,1_ and from *EH*_11_ to *HE*_17,1_, resulting in 18 OAM modes at a telecom wavelength of 1.3 µm. In this numerical design, 2.5 µm was chosen as an optimum ring thickness after performing several simulations because it allows us to remove the modes, i.e., no solution for the field, with a higher radial order (*m* > 1) that is easily coupled with modes of the first radial order (*m* = 1), reducing the number of available OAM modes. In addition, according to [14], most of the OAM modes have a stable behavior. It was found out that OAM modes with |4| from 2 up to 17 are 64 OAM stable states, because its effective index difference ∆*n_e f f_* is around 10^−4^, as indicated by the dashed line in Figure 3.

**Figure 4:**
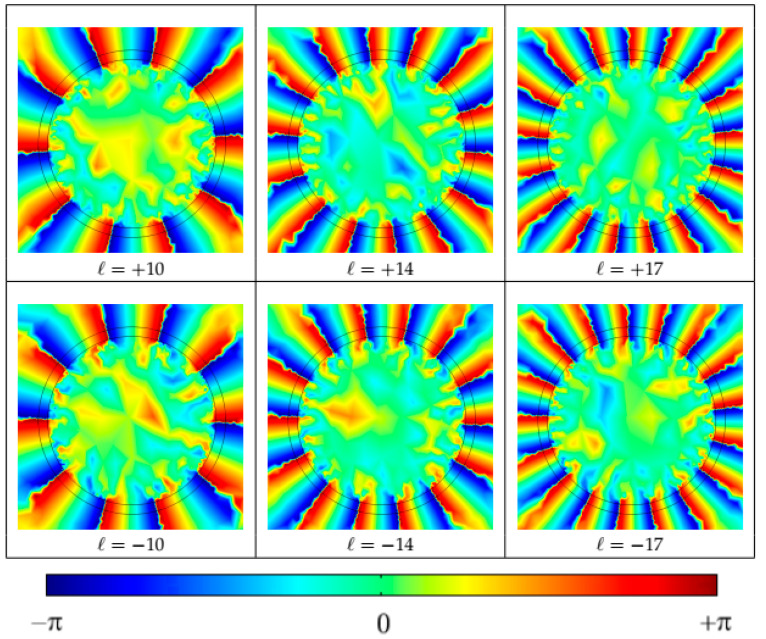
Phase distribution of OAM modes propagated in the simulated vortex-POF. Each SAM σ = ±1 produced two possible states for each OAM mode, yielding, in our case, up to 64 stable OAM states.

The spatial phase distribution given by the term e ^±*il*φ^ for stable OAM modes with *l* = ±10, ±14 and ±17 was also computed (see Figure 4). The spatial phase distributions repeating each 2π (from −π to π) indicate the presence of a component of momentum (*p*) in azimuthal direction (φ), responsible for the orbital angular momentum and the propagation of OAM modes.

### 4.2. Dependence of the Effective Index Difference with the Wavelength of Light

**Figure 5:**
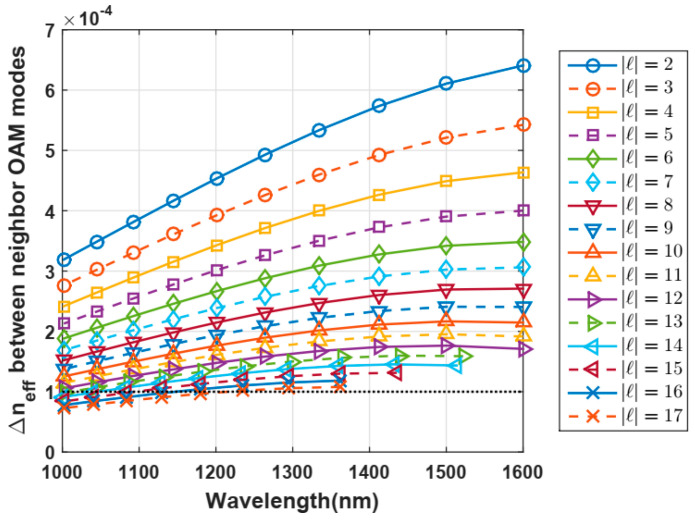
Effective index difference between neighbor OAM modes as a function of light wavelength for the first 18 modes.

Figure 5 shows the OAM modes propagated for telecom wavelengths from 1 to 1.6 µm. It was noticed that our proposed vortex-POF propagates OAM modes with only one radial order *m* = 1 due to the smaller ring size of 2.5 µm with respect to the core size of 50 µm, which is very suitable to combine WDM and OAM multiplexing in fiber-based optical communication systems, in which OAM modes with a radial higher order should be avoided.

The chromatic dispersion through Equation (6) [29] was also computed by means of numerical derivate performed in MATLAB:

(6) $$D=\frac{\lambda }{c}\frac{d^{2}R\left(n_{eff}\right)}{d\lambda^{2}}$$

**Figure 6:**
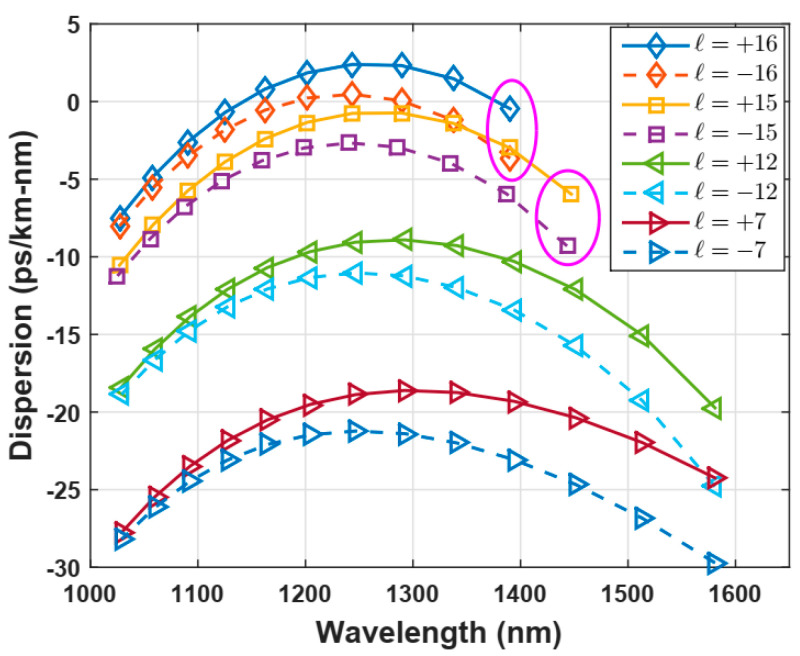
Chromatic dispersion for OAM modes of different topological charges. Circles in magenta indicate the modal cut-off of these OAM modes.

The material dispersion comes from the Sellmeier formula [5], and waveguide dispersion is computed through the second derivate of the effective index as a function of light wavelength. Dispersion curves for some OAM modes are shown in Figure 6. Higher order modes, for example *±l* = −15, +15, −16, +16, possess dispersion values from −2.5 to +2.5 ps/nm-km at 1.3 µm, which is reasonable to achieve transmissions without impairments in fibers of short-range scenarios, according to the proposal of this work (see Figure 6).

**Figure 7:**
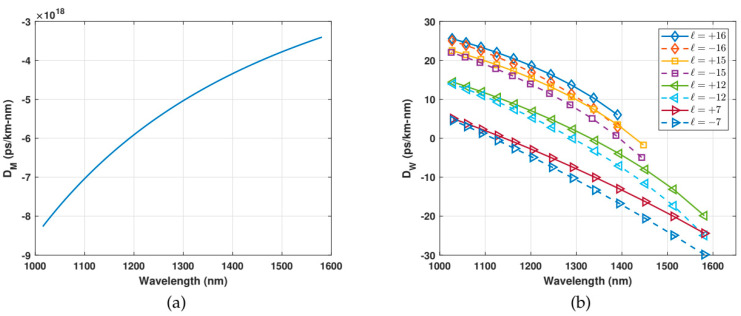
(**a**) Material dispersion. (**b**) Waveguide dispersion

These values can be explained by analyzing the individual contributions to the chromatic dispersion: waveguide dispersion and material dispersion. It can be observed that they are opposite in signs for higher order modes and equal in signs for lower order modes (see Figure 7), giving convenient results for higher order modes.

### 4.3. Propagation Loss

The propagation losses of the OAM modes were computed from the imaginary part of the effective index through Equation (7) [29]:

(7) $$\alpha_{dB/m}=\frac{20}{ln10}\frac{2\pi }{\lambda }I\left(n_{eff}\right)$$

**Figure 8:**
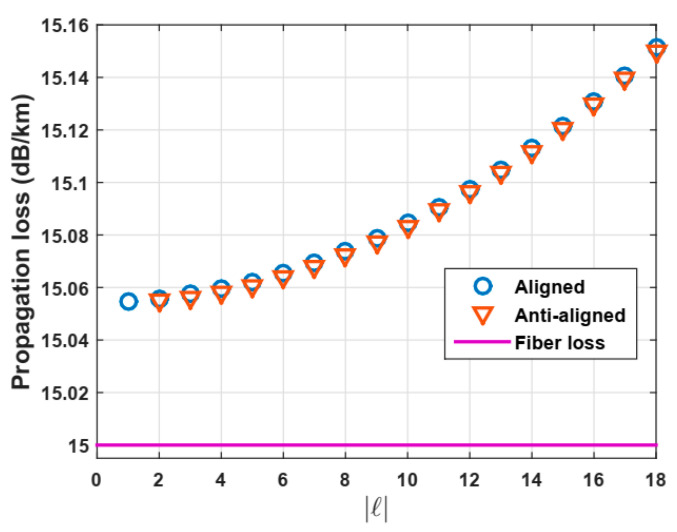
Propagation losses for OAM modes.

Following this approach and considering a wavelength of 1.3 µm, it is observed that the propagation loss of OAM modes remains almost constant with values of approximately 15 × 10^−3^ dB/m, which is almost the same value of the bulk material losses but it can be higher due to impurities and imperfections of boundary layers, such as roughness [7,25], despite modes being confined on the ring in vortex fibers (See Figure 8). Consequently, we also observed that the losses are quite independent of the mode order, so the power of OAM modes can be assumed constant along large propagation fiber lengths. There is a slight increased difference in the propagation loss for higher order modes, i.e., more chance of leakage through the cladding, in which it become evanescent with small bends at this wavelength.

### 4.4. Dependence of the Effective Index Difference with the Ellipticity

We also analyzed the performance of our vortex-POF, considering possible drawbacks in the fabrication process, such as diameter-induced variations along the propagation axis. We considered slight deviations in the circularity (ellipticity) in the diameter with deviations values from 1% up to 5%, which correspond to variations of up to 2.5 µm in one of the transverse axes.

**Figure 9:**
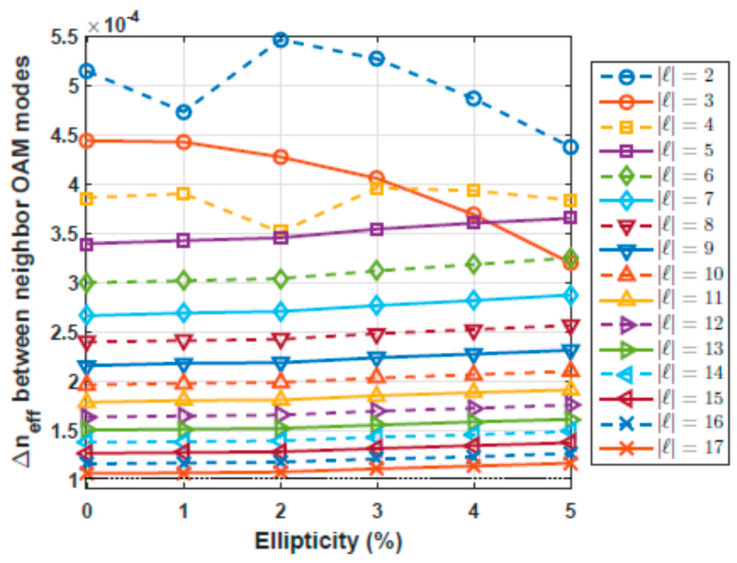
∆*ne f f* as a function of the variations in the circularity of the ring.

Preserving a telecom wavelength of 1.3 µm, we obtained the ∆*n_e f f_* between neighbor OAM modes as a function of the ellipticity, as is shown in Figure 9.

These results show that higher order modes are less affected by variations in the diameter. It can be used as a criterion to choose a more robust OAM mode to be implemented in an MDM-based optical communication system.

### 4.5. OAM Spectra

**Figure 10:**
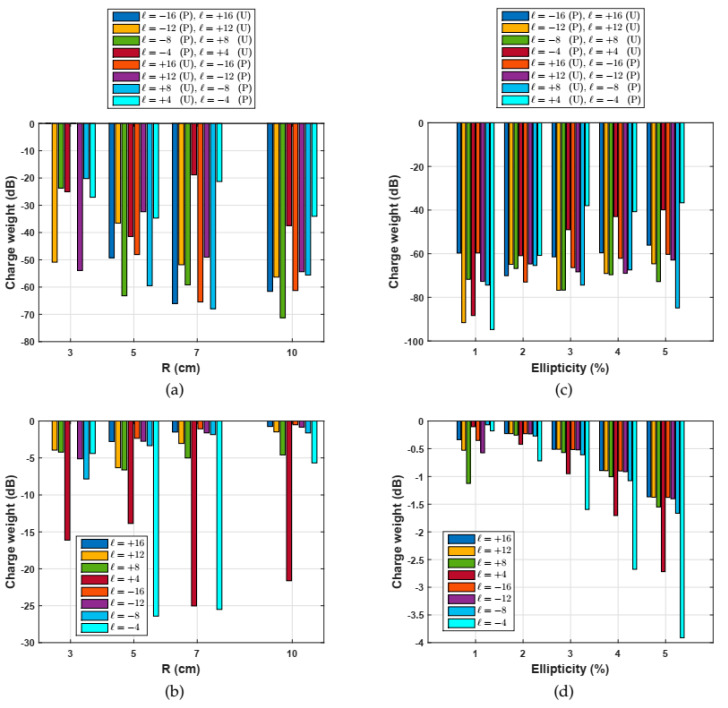
(**Right**) OAM *Spectra* in dB units between some perturbed (P) and unperturbed (U) modes, and considering bending of 3, 5, 7, and 10 cm. (**a**) OAM modes with opposite orbital charges, +*l* and −*l*, of the same value. (**b**) OAM modes with the same orbital charge +*l* or −*l* of the same value. (**Left**) OAM *Spectra* in *dB* units between some perturbed (P) and unperturbed (U) modes and considering ellipticity from 1 up to 5%. (**c**) OAM modes with opposite orbital charge +4 and −4 of the same value. (**d**) OAM modes with the same orbital charge +*l* or −*l* of the same value.

The OAM *spectra* were also obtained by means of the overlap integral of Equation (5), numerically implemented in a MATLAB code using the results of the electric fields of OAM modes coming from COMSOL 5.1. As a first step, the code was tested using unperturbed fields of different orbital charges, assuming a perfectly straight fiber, so values below 120 dB were observed, which means a negligible coupling between the OAM modes, indicating a perfect orthogonality. After that, this integral overlap was implemented by considering perturbations such as small bends and changes in the circularity (ellipticity). Then, Equation (5) was computed using different bend radii from 3 to 10 cm, which are typical values implemented in the fibers deployed around staples or corners. In addition, we also computed Equation (5) by considering slight changes in the circularity from 1% to 5%, which could be defects unavoidable in fabrication processes. Figure 10 shows the results considering bend radius, and Figure 10, ellipticity.

It was noticed that the higher order OAM modes (|*l*| ≥ 12) with opposite orbital charges (+4 and −4) remained quite uncoupled under small bends, exhibiting charge weights lower than −40 dB, but in contrast, the OAM modes of lower order (|*l*| ≤ 4), and also with opposite orbital charges, possessed charge weights around −20 dB, so it can be concluded that higher order OAM modes are more stable. Regarding charge weights between OAM modes with the same orbital charge (+*l* or −*l*) of the same value, high values are observed, which are lower than −10 dB for the higher order OAM modes (|*l*| ≥ 12) and around −20 dB for the lower order OAM modes (|*l|* ≤ 4), showing that the propagation of the OAM modes is not affected by the bends.

Similarly, the same happens with slight ellipticity in the ring diameter, as is shown in Figure 10. The OAM modes with opposite orbital charges remain quite uncoupled under small bends, exhibiting charge weights lower than −40 dB. Regarding charge weights between OAM modes with the same orbital charge, high values of around −4 dB were observed, showing that propagation of the OAM modes is not affected by circularity changes introduced in the fabrication process.

## 5. Conclusions

This research study presented the analysis, by numerical simulations, of a new design of vortex-POF to support up to 64 stable OAM states, with a diameter of 50 µm, making it mechanically compatible with standard POFs at a broad bandwidth from 1 to 1.6 µm. This fiber is proposed as an alternative to be used in short-range optical communication systems or even optical sensors. This vortex-POF shows a difference in effective index between neighbor OAM modes of around 10^−4^ over a bandwidth from 1 to 1.6 µm, a propagation loss of around 15 × 10^−3^ dB/m for all OAM modes, and a dispersion of up to +2.5 ps/km-nm for higher order modes at a telecom wavelength of 1.3 µm. It was also demonstrated that OAM modes in this designed fiber are very stable under perturbations such as bends and slight changes in circularity depending on the optical field. These properties can be exploited in many applications, using this vortex-POF in optical communication and sensors. Although practical realization of this proposed vortex fiber could have higher losses due to impurity or imperfection in the fabrication process, this could have advantages in terms of its performance and easy handling, leading to reduced maintenance and deployed cost of optical communication systems compared with silica fiber.
